# Circulating Markers Reflect Both Anti- and Pro-Atherogenic Drug Effects in ApoE-Deficient Mice

**DOI:** 10.4137/bmi.s632

**Published:** 2008-03-12

**Authors:** Birong Liao, Eileen McCall, Karen Cox, Chung-Wein Lee, Shuguang Huang, Richard E Higgs, Li-Chun Chio, Eugene Zhen, John E Hale, Nancy K Jackson, Pamela G Rutherford, Xiao-di Huang, Donetta Gifford-Moore, Kwan Hui, Kevin Duffin, Kenneth E Gould, Mark Rekhter

**Affiliations:** 1 Integrative Biology; 2 Atherosclerosis and Metabolic Syndrome Drug Hunting Team; 3 Discovery Statistics, Lilly Research Laboratories, Eli Lilly and Company, Indianapolis, IN 46285

## Abstract

**Background:**

Current drug therapy of atherosclerosis is focused on treatment of major risk factors, e.g. hypercholesterolemia while in the future direct disease modification might provide additional benefits. However, development of medicines targeting vascular wall disease is complicated by the lack of reliable biomarkers. In this study, we took a novel approach to identify circulating biomarkers indicative of drug efficacy by reducing the complexity of the in vivo system to the level where neither disease progression nor drug treatment was associated with the changes in plasma cholesterol.

**Results:**

ApoE−/− mice were treated with an ACE inhibitor ramipril and HMG-CoA reductase inhibitor simvastatin. Ramipril significantly reduced the size of atherosclerotic plaques in brachiocephalic arteries, however simvastatin paradoxically stimulated atherogenesis. Both effects occurred without changes in plasma cholesterol. Blood and vascular samples were obtained from the same animals. In the whole blood RNA samples, expression of MMP9, CD14 and IL-1RN reflected pro-and anti-atherogenic drug effects. In the plasma, several proteins, e.g. IL-1β, IL-18 and MMP9 followed similar trends while protein readout was less sensitive than RNA analysis.

**Conclusion:**

In this study, we have identified inflammation-related whole blood RNA and plasma protein markers reflecting anti-atherogenic effects of ramipril and pro-atherogenic effects of simwastatin in a mouse model of atherosclerosis. This opens an opportunity for early, non-invasive detection of direct drug effects on atherosclerotic plaques in complex in vivo systems.

## Introduction

Current drug therapy of atherosclerosis is focused on treatment of major risk factors, e.g. hypercholesterolemia while in the future direct disease modification might provide additional benefits.^1^,^2^ However, discovery and development of medicines targeting vascular wall disease (and hence not inducing any changes of plasma lipids) is complicated by the lack of reliable biomarkers.^2^,^3^ Recent clinical data suggest detrimental cumulative cardiovascular effects of several compounds that improve atherosclerosis risk factors.^4^,^5^ Therefore, early indication of pro-atherogenic drug activities would be desired. To date, vascular imaging remains the only available option.^3^ However, it is expensive and, for some techniques, invasive, that limits its application on a large scale.

Is it possible to find a circulating non-lipid marker that would reflect drug-induced changes in atherosclerotic plaques? In the clinic, correlation between markers of inflammation and risk of cardiovascular events is established.^6^ However, it is unknown whether any of those markers reflects drug-induced changes in the plaque size.

In ApoE-deficient mice, genetically determined hypercholesterolemia leads to development of atherosclerotic lesions.^7^ Importantly, the lesions develop progressively over time while plasma cholesterol levels stay constant.^8^ Therefore, this model provides an opportunity to focus on circulating markers that would be associated with the changes in the plaque size but not in plasma cholesterol. Apparently, this approach is limited by multiple confounding factors such as age and systemic inflammation. However, mere separation of plasma cholesterol and vascular drug effects represents the first important step in unraveling very complex interactions between drug effects and potential blood markers. It has been previously reported that angiotensin-II converting enzyme inhibitor ramipril(Ram) significantly reduced atherosclerotic burden ApoE−/− mice and HMG-CoA reductase inhibitor simvastatin(Sim) paradoxically increased lesion size.^9^–12 In this paper, we decided to exploit this phenomenon and seek circulating markers of cholesterol-independent, drug-induced vascular changes in ApoE-deficient mice. Blood and vascular samples were obtained from the same animals for histology, blood chemistry, blood RNA and protein assessments. The changes in blood chemistry, RNA and protein were correlated with changes in vascular histology.

## Materials and Methods

### Animal experiments

All activities were conducted at Taconic Biotechnology (1 University Place, Rensselaer NY 12144). Apolipoprotein E deficient (ApoE−/−) mice were maintained under murine pathogen free barrier conditions for a duration of 40 weeks with continuous health monitoring, and manipulations were performed with IACUC approved procedures. Animals were fed with Chow diet and maintained at 12-hr light and 12-hr dark cycle. Three to four animals per cage (3 cages/treatment group, 10 animals total) were housed in solid bottom poly-propylene cages with sterilized bedding. At the age of 8 weeks, the mice were either kept on chow diet as control group or treated with ramipril or simvastatin. Ramipril was dissolved in sterilized drinking water and mice were fed at the dose of 5 mg/kg/day for 8, 16 and 24 weeks before sacrifice. Simvastatin was placed in chow diet, mice were fed at the dose of 50 mg/kg/day for 8, 16, 24 weeks before sacrifice. Weekly food and water consumption was recorded for each cage. Two batches of dosing experiments were conducted, one batch of mice were sacrificed for RNA analyses, whereas another batch for protein analysis.

Blood was collected via cardiac puncture procedure. 250 μl of the whole blood was placed directly into the RNA lysis buffer-containing tubes provided by the Source Precision Medicine and frozen. 200 μl of blood was placed in citrate tubes, and plasma was used for protein and lipid analysis.

Immediately following the blood collection, mice were perfused with saline and then IHC Zink fixative via left ventricle. Brachiocephalic artery was dissected, fixed in IHC Zink fixative and paraffin embedded.

### Histology

10 equally spaced (200 μm) paraffin cross sections of the brachiocephalic artery were stained using hematoxylin and eosin. Macrophages and SMC were visualized immunohistochemically using MAC-2 (Accurate Chemical, Westbury, NY) and anti-α-smooth muscle actin antibody (DAKO) respectively. The lesion, defined as an area between the lumen and internal elastic lamina (IEL) was calculated using Image-Pro Plus Version 5.0.1

### Blood analysis

***Plasma lipids*** were analyzed on a Hitachi 912 clinical chemistry analyzer. Total and differential blood cell count was performed by the LabCorps.

#### Quantification of RNA expression

Two hundred fifty μl of whole blood from each animal were sent to Source Precision Medicine (Boulder, CO). Expression of blood mRNA was quantified using its proprietary precision technology (a modified ΔΔCT method) with its validated rodent primers of ABCA1, CD14, HMOX1, HSPA1A, ILLRN, MMP-9, TGFβ1, TLR4, TNFSF5.

#### Immunoassay of plasma inflammation markers

Fifty μl of plasma from each animal were sent to Rules Based Medicine (Austin, TX) and were profiled using its proprietary multiplex assay platform with multiple analyte panel (MAP) version 1.5. The MAP contains 59 molecules.

### Data analysis

Statistical analyses and pattern analyses were performed on SAS, JMP (SAS Institute, NC), MatLab (The MathWorks, MA) and Microsoft Excel.

## Results

### Atherosclerotic plaques developed over time, drug treatment either promoted or repressed atherogenesis

**Figure 1:**
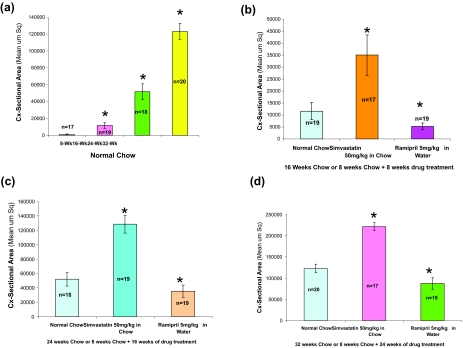
Atherosclerotic plaque develops steadily when ApoE−/− mouse ages from 8 to 32 weeks (**a**). Simvastatin treatment significantly increases plaque formation in ApoE−/− mice, whereas ramipril significantly decreases plaque formation during 8 week (**b**), 16 week (**c**) and 24 week (**d**) treatments. Asterisk indicates p-value of <0.05 compared to the control.

**Figure 2:**
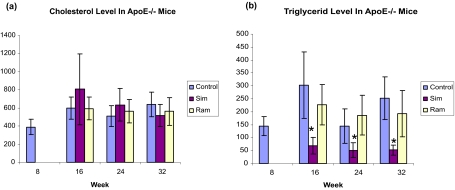
Both simvastatin and ramipril treatments do not alter cholesterol level of ApoE−/− mice. Ramapril treatment does not change the triglyceride level of ApoE−/− mice, whereas simvastatin treatment significantly reduces the triglyceride level of ApoE−/− mice. Asterisk indicates a statistically significant difference with p value <0.05 compared to the same age control.

At the age of 8 weeks, ApoE−/− mice were treated continuously with angiotensin-II converting enzyme inhibitor ramipril, or HMG-CoA reductase inhibitor simvastatin, or by Chow diet alone for 8, 16 and 24 weeks before sacrifice. Figure 1a showed that plaque developed steadily from 8 weeks to 32 weeks in ApoE−/− mice fed with Chow diet. Ramipril treatment significantly reduced the size of plaques compared to the Chow fed mice in all time points sampled (plaque size reduced 55.6%, 31.9% and 28.9% in 8, 16, 24 week treatments with p values of 0.005, 0.027 and 0.004, respectively. Fig. 1b, 1c and 1d); on the contrary, simvastatin increased the atherosclerotic plaques compared to the chow fed mice (plaque size increased 3.0, 2.48 or 1.8 folds respectively, with p values of 0.001). Both treatments had no significant effects on plasma cholesterol (Fig. 2 and Table 1a). Ramipril did not alter the concentration of triglycerids, whereas simvastatin significantly reduced the triglyceride concentration (Fig. 2 and Table 1b).

### Expression of CD14, IL1RN and MMP-9 RNA in whole blood correlated with the effect of ramipril and simvastatin on plaque size

**Figure 3:**
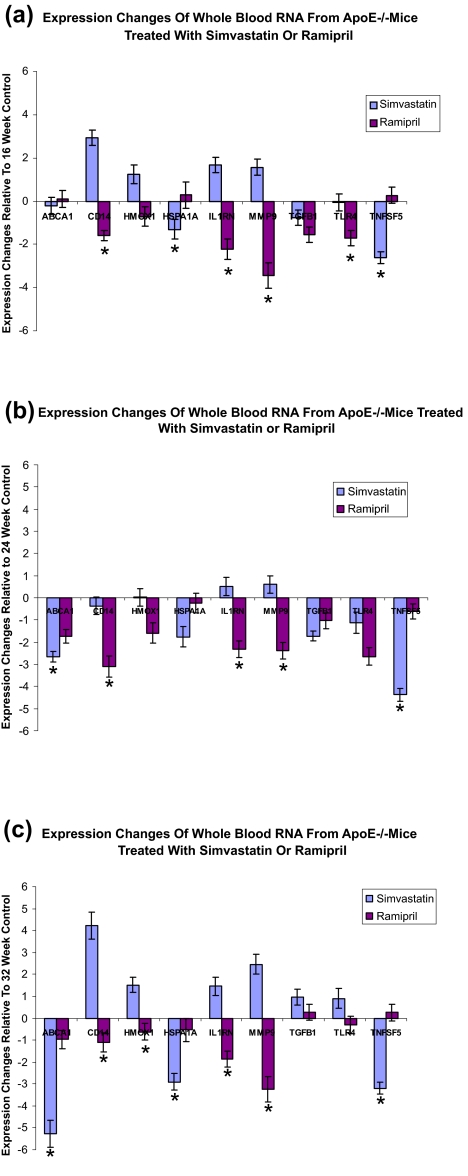
Expression changes of whole blood RNA from simvastatin or ramipril treated ApoE−/− mice at the age of 16 weeks (**a**), 24 weeks (**b**) and 32 weeks (**c**). Y-axis is the fold change compared to the control group (1 means no change). Note the direction and robustness of changes of CD14, IL1RN and MMP9 over treatment times. The mice were initially fed with the Chow diet for 8 weeks before subsequent drug treatments. Asterisk indicates a statically significant difference with p value < 0.05 between simvastatin and ramipril groups.

Expression of nine genes was measured in whole blood of ApoE −/− mice. These RNA were chosen because of available validated rodent primers by Source Precision Medicine (Boulder, CO). Significant differences in expression of CD14, IL1RN and MMP-9 were observed between simvastatin-treated and ramipril-treated ApoE−/− mice (Fig. 3 a–c). Compared to those in the Chow-fed animals, expression of these molecules increased in simvastatin-treated animals whose plaque sizes significantly increased over controls. In contrast, expression of these molecules decreased in ramipril-treated animals (Fig. 3 a–c) whose plaque size was significantly reduced compared to the Chow-fed controls. The data suggest that CD14, IL1RN and MMP-9 are good biochemical markers of drug effects on atherosclerotic lesion size. Furthermore, the difference in markers can be measured as early as 8 weeks after the beginning of drug treatment (Fig. 3a).

### Concentration of pro-inflammatory molecules fibrinogen, IL-1b, IL-18, M-CSF, MMP-9, CD40 and VCAM-1 in plasma correlated with the effect of ramipril and simvastatin on plaque size

**Figure 4:**
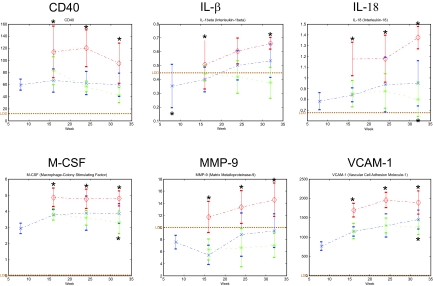
The plasma concentration of CD14, fibrinogen, IL1β, IL18, MCSF, MMP-9 and VCAM-1 changes in ApoE−/− mice treated with simvastatin or ramipril. In general, the concentrations of these markers increase in mice treated with simvastatin, whereas they decrease in mice treated with ramipril. LDD—Least Detectable Dose as determined by Rules Based Medicine (Austin, TX), red line—simvastatin treatment, green line—ramipril treatment, blue line—the Chow fed control. Asterisk indicates a statically significant difference with p value < 0.05 between simvastatin/or ramipril and control groups.

Plasma samples were analyzed on multiplex assay platform (MAP version 1.5) by Rules Based Medicine (Austin, TX). These molecules and platform were chosen because of available validated rodent antibodies by RBM. Out of 59 molecules analyzed, the concentration of IL-1b, IL-18, M-CSF, MMP-9, CD40 and VCAM-1 increased in simvastatin-treated mice, decreased in ramipril-treated mice (Fig. 4, Appendix). Furthermore, the changes of these molecules in simvastatin-treated mice comparing to the controls are much more profound than the changes in ramipril-treated mice comparing to the controls. The result suggests that these molecules are potential biomarkers of drug effects on atherosclerotic lesion size, at least in the case of pro-atherogenic changes. The difference in the concentration of markers can be measured as early as 16 weeks after the beginning of drug treatment for IL-1b and MMP-9; all can be measured at 24 weeks after the beginning of drug treatment (Fig. 4).

### No difference in white blood cell number among different treatment groups

To dissect if the difference in gene expression and concentration of protein markers were affected by the cell number change, number of white blood cell from all treatment groups was counted (Table 2), no statistically significant difference among the groups were found.

## Discussion

In this paper, we have identified the non-lipid circulating biomarkers indicative of both anti- and pro-atherogenic drug effects. We treated ApoE−/− mice with an ACE inhibitor ramipril and HMG-CoA reductase inhibitor simvastatin. Ramipril significantly reduced the size of atherosclerotic plaques in brachiocephalic arteries, however simvastatin paradoxically stimulated atherogenesis. Both effects occurred without changes in plasma cholesterol. We decided to exploit this phenomenon and seek circulating markers of cholesterol-independent, drug-induced vascular changes in ApoE-deficient mice. Blood and vascular samples were obtained from the same animals. In the whole blood RNA samples, expression of MMP9, CD14 and IL-1RN reflected pro-and anti-atherogenic drug effects. In the plasma, several proteins, e.g. IL-1β, IL-18 and MMP9 followed similar trends while protein readout was less sensitive than RNA analysis.

Ramipril is the only ACE inhibitor that is currently approved for the prevention of cardiovascular events in high risk patients based on the results of the HOPE trial.^13^ It was demonstrated that in the normotensive patients, cardiovascular benefits of ramipril are independent of its blood pressure lowering effects.^14^ ACE inhibitors also do not have lipid-lowering properties. Experimental data strongly suggest that these effects of ACE inhibitors are mediated by their direct anti-inflammatory activity that ameliorates pro-inflammatory signaling of angiotensin II in the vasculature.^15^ Specifically, ramipril attenuated atherosclerosis in ApoE−/− mice in a blood pressure- and cholesterol-independent manner^10^ while preventing macrophage activation.^9^ In agreement with these data, we demonstrated that ramipril treatment significantly reduced atherosclerotic plaque size in ApoE−/− mice. Moreover, we have identified several blood markers that have changed accordingly.

Statins, including simvastatin, have unequivocal effect on the reduction of cardiovascular and all-cause mortality, and this is likely due to the reduction in cholesterol.^16^ However, the data on statin efficacy in ApoE−/− mice are controversial.^17^ Sparrow et al. reported anti-inflammatory and anti-atherosclerotic activities of simvastatin exerted without any changes of plasma lipids.^18^ Short-term anti-inflammatory effects are also documented in the study of Scalia et al.^19^ However, several groups reported significant elevation of plasma cholesterol associated with increased atherosclerotic burden^20^ and plaque size^12^ in the mice with spontaneous atherosclerosis or intimal hyperplasia induced by mechanical injury of the artery.^11^ It is suggested that paradoxical plasma cholesterol elevation could be driven by formation of cholesterol-rich remnants in apoE−/− mice.^21^ Overall, pro-atherogenic effects of simvastatin in these studies are assumed to be the consequence of cholesterol elevation.

Our results, however, demonstrated an increase in plaque size without significant plasma cholesterol elevation. That suggests potential direct pro-atherogenic (likely pro-inflammatory) vascular effects of the drug in this animal model. Albeit counter-intuitive, this mechanism is supported by the data obtained in cultured human monocyte-derived macrophages. Kiener et al. reported that simvastatin stimulated production of MCP-1, IL-8, IL-1β and TNF-α. 22 Lindholm and Nilsson recently demonstrated that simvastatin stimulated IL-1β secretion.^23^ Simvastatin also exerted pro-inflammatory effects in a mouse model of peritonitits.^22^ Recently, direct pro-apoptotic effects of simvastatin in human endothelial cells have been demonstrated.^24^ Thus, it is plausible that under certain experimental circumstances simvastatin may exhibit pro-inflammatory properties that in the clinic either do not occur or are counterbalanced by profound lipid lowering thereby providing ultimate therapeutic benefits. In the current study, we have not attempted detailed mechanistic analysis of simvastatin activity in ApoE−/− mice. Rather, we capitalized on the observation that its pro-atherogenic effects, regardless the mechanism, were associated with an upsurge of several blood inflammation markers.

Taken together, ramipril and simvastatin arms of the study demonstrated that the same set of circulating markers responded in coordinated manner to both anti- and pro-atherogenic, lipid-independent drug effects. Exact mechanistic links between vascular effects of these drugs and their effects on the circulating markers of inflammation are unknown. Several possibilities and combinations thereof exist. Moreover, the genesis of blood RNA and protein markers is likely to be different.

Whole blood RNA represents gene expression of various circulating cell populations, predominantly white blood cells, although it is impossible to exclude a contribution of red blood cells and platelets. White blood cell count per se may be associated with atherosclerosis.^25^ That phenomenon alone might be responsible for apparent changes in gene expression. Our data though demonstrated no drug effect on the white blood cell number. However, enrichment of specific leukocyte types may still account for the RNA changes. It has been shown that the rise in monocyte count is associated with plaque formation in humans^26^ and in the Western diet-fed ApoE−/− mice.^27^ In the current study, we have not detected any significant changes in monocyte numbers. However, more detailed analysis of monocyte sub-populations is granted. Thus, our data suggest that both ramipril and simvastatin could exert effects on the gene expression rather than affect the cell number. It remains unclear whether the drugs directly affected gene expression in circulating cells or, alternatively, blood RNA changes were secondary to the vascular wall effects. This is a fundamental and yet unanswered question that demands future in-depth research. The answer will determine how blood RNA changes will be positioned, i.e. as pharmacodynamic markers of drug activity or as circulating markers reflecting biology of atherosclerotic plaques.

Specific tissue sources of plasma protein changes are even less clear. MMPs and interleukins (the proteins that, according to our data, seem to be sensitive to ramipril and simvastatin treatment) could originate, among other tissues, in the liver, adipose tissue or atherosclerotic plaques themselves. Regardless the exact origin, however, they have potential to become useful markers assuming that described effects can be extended to the other drugs capable of modifying vascular wall.

Although only limited set of genes and proteins was analyzed, it is tempting to speculate about coordinated nature of identified changes. Noticeable MMP9 dynamics (downward with ramipril and upward with simvastatin) was detected both at the level of blood RNA and plasma protein, suggestive of the multiple tissues response. MMP9 protein levels in the lesions and in plasma are associated with plaque development and rupture in human and mouse atherosclerosis.^28^–^30^ Extending that knowledge to circulating cell RNA (far more sensitive readout in this study) and demonstration of drug effects further validates this marker and potentially increases it’s utility. CD14 gene up-regulation in circulating leukocytes is consistent with emerging role of innate immunity in atherosclerosis.^1^ It is likely that plasma IL1 protein elevation reflects one of the downstream effects of activated innate immunity signaling. IL-1RN up-regulation, anti-inflammatory by nature, may indicate a negative compensatory feedback response to IL-1 elevation.^31^ In this case, an anti-inflammatory gene may be paradoxically portrayed as a sensitive marker of inflammation and/or atherosclerosis.

We had also attempted to use whole blood proteomic approach (data not shown). However, whole blood proteomics has yet to provide discriminatory power that was necessary to identify the changes in inflammatory protein molecules. A detailed report on comparing the whole blood shotgun proteomics vs. targeted proteomics approaches will be presented elsewhere.

In aggregate, our data suggest that a set of inflammation-related markers, both at the level of circulating leukocyte activation and systemic response may be indicative of pro- and anti-atherogenic drug effects. Current study represents the first step towards identification of circulating markers reflecting lipid-independent, disease-modifying drug effects. The number of compounds with different mechanism of action as well as candidate genes and proteins needs to be extended. If further validated, presented approach might be useful in early prediction of vascular efficacy and/or potential vascular toxicity of investigational drugs.

## Figures and Tables

**Table 1:** Cholesterol (**a**) and Triglyceride (**b**) concentration in ApoE−/− Mice.

(**a**)				
Week	8	16	24	32
Control	389.75 + 83.74	599.5 + 122.30	511.32 + 114.87	640.00 + 135.15
Simvastatin		806.18 + 392.78	633.89 + 180.28	516.39 + 119.91
Ramipril		594.25 + 126.12	564 + 131.54	562.00 + 151.51

(**b**)				
Week	8	16	24	32
Control	145.00 + 36.09	302.75 + 128.86	145.53 + 66.58	252.00 + 82.90
Simvastatin		68.82 + 32.19	51.58 + 29.58	51.94 + 19.64
Ramipril		226.50 + 78.46	186.75 + 77.28	192.00 + 89.02

**Table 2:** White Blood Cell Count From ApoE−/− Mice*.

Week	Treatment	WBC (10^3/μL)
32	Control	3.08 ± 0.70
32	Simvastatin	3.35 ± 1.66
32	Ramipril	2.67 ± 0.98
24	Control	2.96 ± 0.66
24	Simvastatin	1.69 ± 0.53
24	Ramipril	2.70 ± 0.63
16	Control	1.85 ± 0.84
16	Simvastatin	1.97 ± 0.15
16	Ramipril	NA
8	Control	2.05 ± 0.92

* There is no statistical significant difference among groups.

## Appendix

**Table:** Multi-analyte profile of plasma protein from apoE−/− mice.

Marker	Treatment1^a^	Treatment2^b^	Conc_1	Conc_2	Fold change	p value	LDD^c^
CD40	16wRam	16wCon	82.81 ± 22.949	66.74 ± 19.237	1.24	0.1284	12
CD40	16wSim	16wCon	113.84 ± 42.431	66.74 ± 19.237	1.71	0.0001	12
CD40	24wRam	24wCon	56.96 ± 8.867	62.41 ± 19.322	−1.1	0.6445	12
CD40	24wSim	24wCon	119.97 ± 30.577	62.41 ± 19.322	1.92	0	12
CD40	32wRam	32wCon	42.5 ± 12.481	59.58 ± 19.073	−1.4	0.1159	12
CD40	32wSim	32wCon	95.33 ± 33.306	59.58 ± 19.073	1.6	0.0033	12
CD40	16wCon	8wCon	66.74 ± 19.237	59.81 ± 9.015	1.12	0.5208	12
CD40	16wRam	8wCon	82.81 ± 22.949	59.81 ± 9.015	1.38	0.0354	12
CD40	16wSim	8wCon	113.84 ± 42.431	59.81 ± 9.015	1.9	0	12
CD40	24wCon	8wCon	62.41 ± 19.322	59.81 ± 9.015	1.04	0.8257	12
CD40	24wRam	8wCon	56.96 ± 8.867	59.81 ± 9.015	−1.1	0.7963	12
CD40	24wSim	8wCon	119.97 ± 30.577	59.81 ± 9.015	2.01	0	12
CD40	32wCon	8wCon	59.58 ± 19.073	59.81 ± 9.015	−1	0.9832	12
CD40	32wRam	8wCon	42.5 ± 12.481	59.81 ± 9.015	−1.4	0.1111	12
CD40	32wSim	8wCon	95.33 ± 33.306	59.81 ± 9.015	1.59	0.0035	12
Fibrinogen	16wRam	16wCon	3358 ± 949.18	3810 ± 500.58	−1.1	0.398	12
Fibrinogen	16wSim	16wCon	3615 ± 496.47	3810 ± 500.58	−1.1	0.7305	12
Fibrinogen	24wRam	24wCon	4037.78 ± 646.43	4596.67 ± 1647.3	−1.1	0.3218	12
Fibrinogen	24wSim	24wCon	3408.89 ± 744.32	4596.67 ± 1647.3	−1.3	0.0372	12
Fibrinogen	32wRam	32wCon	3614 ± 648.08	4371 ± 971.3	−1.2	0.1585	12
Fibrinogen	32wSim	32wCon	5494.29 ± 3060.4	4371 ± 971.3	1.26	0.0588	12
Fibrinogen	16wCon	8wCon	3810 ± 500.58	2553.33 ± 754.4	1.49	0.0241	12
Fibrinogen	16wRam	8wCon	3358 ± 949.18	2553.33 ± 754.4	1.32	0.1448	12
Fibrinogen	16wSim	8wCon	3615 ± 496.47	2553.33 ± 754.4	1.42	0.0699	12
Fibrinogen	24wCon	8wCon	4596.67 ± 1647.3	2553.33 ± 754.4	1.8	0.0005	12
Fibrinogen	24wRam	8wCon	4037.78 ± 646.43	2553.33 ± 754.4	1.58	0.0097	12
Fibrinogen	24wSim	8wCon	3408.89 ± 744.32	2553.33 ± 754.4	1.34	0.1309	12
Fibrinogen	32wCon	8wCon	4371 ± 971.3	2553.33 ± 754.4	1.71	0.0013	12
Fibrinogen	32wRam	8wCon	3614 ± 648.08	2553.33 ± 754.4	1.42	0.0558	12
Fibrinogen	32wSim	8wCon	5494.29 ± 3060.4	2553.33 ± 754.4	2.15	0	12
IL_18	16wRam	16wCon	0.85 ± 0.122	0.84 ± 0.065	1.01	0.8771	0.67
IL_18	16wSim	16wCon	1.18 ± 0.155	0.84 ± 0.065	1.4	0.0001	0.67
IL_18	24wRam	24wCon	0.88 ± 0.161	0.94 ± 0.254	−1.1	0.4334	0.67
IL_18	24wSim	24wCon	1.18 ± 0.218	0.94 ± 0.254	1.26	0.0027	0.67
IL_18	32wRam	32wCon	0.8 ± 0.161	0.95 ± 0.21	−1.2	0.0475	0.67
IL_18	32wSim	32wCon	1.37 ± 0.103	0.95 ± 0.21	1.45	0	0.67
IL_18	16wCon	8wCon	0.84 ± 0.065	0.78 ± 0.079	1.07	0.4435	0.67
IL_18	16wRam	8wCon	0.85 ± 0.122	0.78 ± 0.079	1.09	0.3598	0.67
IL_18	16wSim	8wCon	1.18 ± 0.155	0.78 ± 0.079	1.5	0	0.67
IL_18	24wCon	8wCon	0.94 ± 0.254	0.78 ± 0.079	1.2	0.0498	0.67
IL_18	24wRam	8wCon	0.88 ± 0.161	0.78 ± 0.079	1.12	0.2319	0.67
IL_18	24wSim	8wCon	1.18 ± 0.218	0.78 ± 0.079	1.5	0	0.67
IL_18	32wCon	8wCon	0.95 ± 0.21	0.78 ± 0.079	1.21	0.0317	0.67
IL_18	32wRam	8wCon	0.8 ± 0.161	0.78 ± 0.079	1.02	0.8209	0.67
IL_18	32wSim	8wCon	1.37 ± 0.103	0.78 ± 0.079	1.75	0	0.67
IL_1beta	16wRam	16wCon	0.44 ± 0.055	0.4 ± 0.089	1.11	0.3863	0.45
IL_1beta	16wSim	16wCon	0.51 ± 0.175	0.4 ± 0.089	1.27	0.048	0.45
IL_1beta	24wRam	24wCon	0.42 ± 0.118	0.5 ± 0.105	−1.2	0.1166	0.45
IL_1beta	24wSim	24wCon	0.61 ± 0.093	0.5 ± 0.105	1.21	0.0519	0.45
IL_1beta	32wRam	32wCon	0.37 ± 0.111	0.54 ± 0.12	−1.4	0.002	0.45
IL_1beta	32wSim	32wCon	0.66 ± 0.042	0.54 ± 0.12	1.23	0.026	0.45
IL_1beta	16wCon	8wCon	0.4 ± 0.089	0.35 ± 0.156	1.13	0.3782	0.45
IL_1beta	16wRam	8wCon	0.44 ± 0.055	0.35 ± 0.156	1.26	0.0916	0.45
IL_1beta	16wSim	8wCon	0.51 ± 0.175	0.35 ± 0.156	1.44	0.0075	0.45
IL_1beta	24wCon	8wCon	0.5 ± 0.105	0.35 ± 0.156	1.42	0.0077	0.45
IL_1beta	24wRam	8wCon	0.42 ± 0.118	0.35 ± 0.156	1.18	0.236	0.45
IL_1beta	24wSim	8wCon	0.61 ± 0.093	0.35 ± 0.156	1.72	0	0.45
IL_1beta	32wCon	8wCon	0.54 ± 0.12	0.35 ± 0.156	1.52	0.0009	0.45
IL_1beta	32wRam	8wCon	0.37 ± 0.111	0.35 ± 0.156	1.06	0.6756	0.45
IL_1beta	32wSim	8wCon	0.66 ± 0.042	0.35 ± 0.156	1.88	0	0.45
MMP_9	16wRam	16wCon	6.33 ± 1.749	5.43 ± 1.606	1.17	0.4204	10
MMP_9	16wSim	16wCon	11.7 ± 2.561	5.43 ± 1.606	2.15	0	10
MMP_9	24wRam	24wCon	6.61 ± 3.155	8.79 ± 3.575	−1.3	0.0669	10
MMP_9	24wSim	24wCon	13.36 ± 2.711	8.79 ± 3.575	1.52	0.0002	10
MMP_9	32wRam	32wCon	7.01 ± 1.989	9.44 ± 2.785	−1.3	0.0316	10
MMP_9	32wSim	32wCon	14.57 ± 2.769	9.44 ± 2.785	1.54	0.0001	10
MMP_9	16wCon	8wCon	5.43 ± 1.606	7.55 ± 1.138	−1.4	0.0668	10
MMP_9	16wRam	8wCon	6.33 ± 1.749	7.55 ± 1.138	−1.2	0.2879	10
MMP_9	16wSim	8wCon	11.7 ± 2.561	7.55 ± 1.138	1.55	0.0009	10
MMP_9	24wCon	8wCon	8.79 ± 3.575	7.55 ± 1.138	1.16	0.2941	10
MMP_9	24wRam	8wCon	6.61 ± 3.155	7.55 ± 1.138	−1.1	0.4252	10
MMP_9	24wSim	8wCon	13.36 ± 2.711	7.55 ± 1.138	1.77	0	10
MMP_9	32wCon	8wCon	9.44 ± 2.785	7.55 ± 1.138	1.25	0.1023	10
MMP_9	32wRam	8wCon	7.01 ± 1.989	7.55 ± 1.138	−1.1	0.6352	10
MMP_9	32wSim	8wCon	14.57 ± 2.769	7.55 ± 1.138	1.93	0	10
M_CSF	16wRam	16wCon	3.8 ± 0.362	3.77 ± 0.327	1.01	0.8943	0.018
M_CSF	16wSim	16wCon	4.86 ± 0.573	3.77 ± 0.327	1.29	0.0002	0.018
M_CSF	24wRam	24wCon	3.59 ± 0.456	3.88 ± 1.067	−1.1	0.2943	0.018
M_CSF	24wSim	24wCon	4.76 ± 0.595	3.88 ± 1.067	1.23	0.0019	0.018
M_CSF	32wRam	32wCon	3.31 ± 0.699	3.85 ± 0.611	−1.2	0.0449	0.018
M_CSF	32wSim	32wCon	4.79 ± 0.467	3.85 ± 0.611	1.25	0.0016	0.018
M_CSF	16wCon	8wCon	3.77 ± 0.327	2.93 ± 0.322	1.29	0.0026	0.018
M_CSF	16wRam	8wCon	3.8 ± 0.362	2.93 ± 0.322	1.3	0.0017	0.018
M_CSF	16wSim	8wCon	4.86 ± 0.573	2.93 ± 0.322	1.66	0	0.018
M_CSF	24wCon	8wCon	3.88 ± 1.067	2.93 ± 0.322	1.32	0.001	0.018
M_CSF	24wRam	8wCon	3.59 ± 0.456	2.93 ± 0.322	1.22	0.0203	0.018
M_CSF	24wSim	8wCon	4.76 ± 0.595	2.93 ± 0.322	1.63	0	0.018
M_CSF	32wCon	8wCon	3.85 ± 0.611	2.93 ± 0.322	1.31	0.0011	0.018
M_CSF	32wRam	8wCon	3.31 ± 0.699	2.93 ± 0.322	1.13	0.1617	0.018
M_CSF	32wSim	8wCon	4.79 ± 0.467	2.93 ± 0.322	1.64	0	0.018
VCAM_1	16wRam	16wCon	1151.8 ± 194.51	1141 ± 117.99	1.01	0.9075	0.95
VCAM_1	16wSim	16wCon	1692.5 ± 176.7	1141 ± 117.99	1.48	0	0.95
VCAM_1	24wRam	24wCon	1296.67 ± 188.75	1296.67 ± 301.04	0	1	0.95
VCAM_1	24wSim	24wCon	1956.67 ± 197.23	1296.67 ± 301.04	1.51	0	0.95
VCAM_1	32wRam	32wCon	1252.6 ± 186.93	1449 ± 243.33	−1.2	0.0371	0.95
VCAM_1	32wSim	32wCon	1891.43 ± 297.96	1449 ± 243.33	1.31	0	0.95
VCAM_1	16wCon	8wCon	1141 ± 117.99	763.22 ± 107.73	1.49	0.0002	0.95
VCAM_1	16wRam	8wCon	1151.8 ± 194.51	763.22 ± 107.73	1.51	0.0001	0.95
VCAM_1	16wSim	8wCon	1692.5 ± 176.7	763.22 ± 107.73	2.22	0	0.95
VCAM_1	24wCon	8wCon	1296.67 ± 301.04	763.22 ± 107.73	1.7	0	0.95
VCAM_1	24wRam	8wCon	1296.67 ± 188.75	763.22 ± 107.73	1.7	0	0.95
VCAM_1	24wSim	8wCon	1956.67 ± 197.23	763.22 ± 107.73	2.56	0	0.95
VCAM_1	32wCon	8wCon	1449 ± 243.33	763.22 ± 107.73	1.9	0	0.95
VCAM_1	32wRam	8wCon	1252.6 ± 186.93	763.22 ± 107.73	1.64	0	0.95
VCAM_1	32wSim	8wCon	1891.43 + 297.96	763.22 ± 107.73	2.48	0	0.95

a,b Week and treatment. For example, 32wSim indicates simvastatin treated animals at the age of 32 weeks. Ram –Ramipril, Con – control.

c Least detectable dose.
